# The Role of Pediatric Nutrition as a Modifiable Risk Factor for Precocious Puberty

**DOI:** 10.3390/life11121353

**Published:** 2021-12-07

**Authors:** Valeria Calcaterra, Elvira Verduci, Vittoria Carlotta Magenes, Martina Chiara Pascuzzi, Virginia Rossi, Arianna Sangiorgio, Alessandra Bosetti, Gianvincenzo Zuccotti, Chiara Mameli

**Affiliations:** 1 Pediatric Department, “Vittore Buzzi” Children’s Hospital, 20154 Milan, Italy; valeria.calcaterra@unipv.it (V.C.); vittoria.magenes@unimi.it (V.C.M.); martina.pascuzzi@unimi.it (M.C.P.); virginia.rossi@unimi.it (V.R.); arianna.sangiorgio@unimi.it (A.S.); alessandra.bosetti@asst-fbf-sacco.it (A.B.); gianvincenzo.zuccotti@unimi.it (G.Z.); chiara.mameli@unimi.it (C.M.); 2 Pediatric and Adolescent Unit, Department of Internal Medicine, University of Pavia, 27100 Pavia, Italy; 3 Department of Health Sciences, University of Milan, 20142 Milan, Italy; 4 Department of Biomedical and Clinical Science “L. Sacco”, University of Milan, 20157 Milan, Italy

**Keywords:** children, diet, nutrients, nutrition, precocious puberty, timing of puberty

## Abstract

Puberty is a critical phase of growth and development characterized by a complex process regulated by the neuroendocrine system. Precocious puberty (PP) is defined as the appearance of physical and hormonal signs of pubertal development at an earlier age than is considered normal. The timing of puberty has important public health, clinical, and social implications. In fact, it is crucial in psychological and physical development and can impact future health. Nutritional status is considered as one of the most important factors modulating pubertal development. This narrative review presents an overview on the role of nutritional factors as determinants of the timing of sexual maturation, focusing on early-life and childhood nutrition. As reported, breast milk seems to have an important protective role against early puberty onset, mainly due to its positive influence on infant growth rate and childhood overweight prevention. The energy imbalance, macro/micronutrient food content, and dietary patterns may modulate the premature activation of the hypothalamic–pituitary–gonadal axis, inducing precocious activation of puberty. An increase in knowledge on the mechanism whereby nutrients may influence puberty will be useful in providing adequate nutritional recommendations to prevent PP and related complications.

## 1. Introduction

Puberty, a critical phase of growth and development, is a complex process that starts with a growth spurt and the development of secondary sex characteristics and culminates in the acquisition of psychophysical maturity and reproductive capacity [1,2].

Precocious puberty (PP) is defined as the appearance of physical and hormonal signs of pubertal development at an earlier age than is considered normal. The onset and progression of puberty are regulated by the neuroendocrine system [2]. Physiologically, the onset of puberty follows the disinhibition of the hypothalamic–pituitary–gonadal axis (HPG), resulting in the progressive amplification of the pulsatile secretion of gonadotropin-releasing hormone (GnRH) by hypothalamic neurons. GnRH stimulates the pulsatile release of luteinizing hormone (LH) and follicle-stimulating hormone (FSH) by the pituitary, inducing the production of sex hormones that are responsible for the physical manifestations of puberty [3]. The timing of puberty has important public health, clinical, and social implications. In fact, the regular timing of puberty is crucial to psychological and physical development and can impact future health [4]. As previously reported, PP has been associated with short adult stature, adverse psychological outcomes, increased risk of obesity, hypertension, type two diabetes, cardiovascular disease, and estrogen-dependent cancer, particularly breast cancer [5].

The interactions between genetic, endocrine, and environmental factors are crucial in pubertal timing [1,2]. Nutritional status is considered one of the most important factors involved in pubertal development [6] and it was estimated to explain as much as 25% of the variation in the timing of puberty [6]. Early-life and childhood nutrition may have an impact on the timing of puberty onset. Several studies have proposed mechanisms by which energy imbalance, macro/micronutrient food content and dietary patterns may modulate the premature activation of the HPG axis, inducing PP.

This narrative review presents an overview on the role of nutritional factors as determinants of the timing of sexual maturation, focusing on early-life and childhood nutrition. An increase in knowledge on the mechanism whereby nutrients may influence puberty will be useful in developing nutritional recommendations to maintain the regular timing of puberty. Adherence to adequate nutritional recommendations in pediatrics may contribute to preventing PP and related complications.

## 2. Methods of Data Collection

A narrative review was performed [7]. The authors independently reviewed relevant English literature on the influence of nutritional status on the timing of puberty in the past 15 years, including original papers, meta-analysis, clinical trials, and reviews. Case reports or series and letters were excluded. A search in PubMed, Scopus, EMBASE, and Web of Science was carried out. Papers published up to September 2021 were searched with the following keywords (alone and/or in combination): precocious puberty, early puberty, timing of puberty, nutrition, diet, micronutrients, macronutrients, breastfeeding, soy-based formulas, formula feeding, complementary feeding. The contributions were critically reviewed and collected by V.C., E.V., V.C.M., M.C.P., V.R., A.S., and M.C. The resulting draft was discussed with all coauthors and the final version was approved by all.

## 3. Discussion of the Literature

### 3.1. Puberty

#### 3.1.1. Physiology of Puberty

Puberty is a major stage in the growth of any human being [1,2]. It is a term that encompasses a complex series of biological phenomena that occur during adolescence. It usually proceeds in a uniform pattern with some variability as to when it occurs, its sequence, and its duration, finally resulting in full reproductive capacity [8]. Therefore, it defines the shift from sexual immaturity to sexual maturity [9]. 

**Figure 1:**
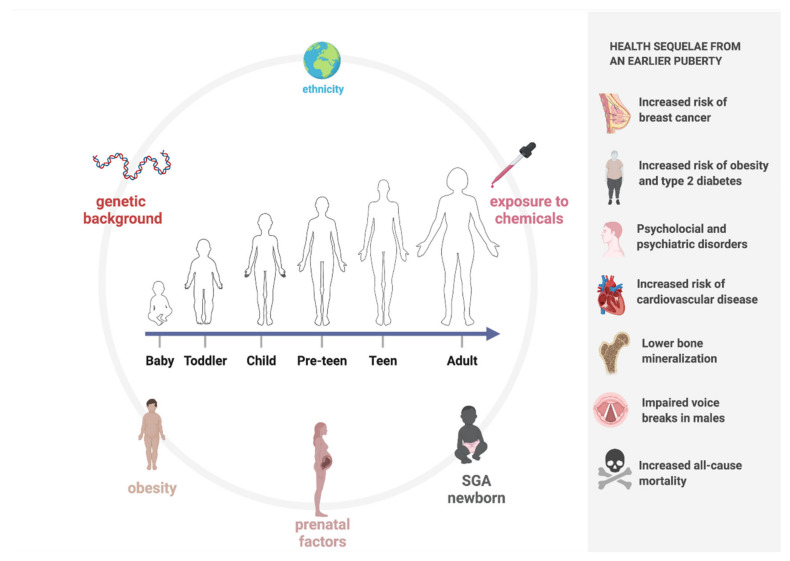
Timing in puberty: factors responsible for lowering and health sequelae from an earlier puberty (created with BioRender.com (accessed on 9 November 2021)).

Pubertal timing is dictated by the interplay and synergism between hormones, central neurotransmitters, and environmental factors that lead to the activation of the HPG axis [1,2], as shown in Figure 1.

This complex dialogue begins from the earliest stages of development [9]: from the first week of life, as the maternal steroid hormones abruptly decline, a phenomenon termed “mini-puberty” occurs, i.e., the initial activation of the HPG axis, leading to the increased synthesis of steroid hormones [1,10]. The increase in LH and FSH triggers the release of testosterone from the testes in males and estradiol from the ovaries in females. The hormonal levels during mini-puberty impact genital organ development, fertility, somatic growth, and body composition during the first 12 months of life, as well as cognitive development [1,2,10]. This phenomenon lasts for approximately the first six months of life, after which the axis remains inactive until five years of age, and it is then finally reactivated during adolescence, when FSH and LH secretion from the adenohypophysis is elicited by gonadotropin-releasing hormone (GnRH) released from the hypothalamus in a pulsatile pattern to avoid the downregulation of its receptor in the hypophysis [1,2,10]. FSH and LH promote spermatogenesis and oogenesis along with testosterone and estradiol release in males and females, respectively [2,11].

The pulsatile stimuli source of GnRH is tightly regulated by both excitatory and inhibitory control; therefore, the excitatory signal rises while the inhibitory signal decreases at the onset of puberty [10]. Several strongly linked stimuli, including leptin, kisspeptin, neurokinin B, and glutamate, along with numerous glial signaling molecules, are involved [2]. The neurons in the arcuate nucleus that release kisspeptin/neurokinin B/dynorphin A (KNDy), along with those that produce inhibitory signals, play a key role in the regulation of pulsatile GnRH secretion [12], resulting in direct feedback to the GnRH pulse generator [2,13]. GnRH secretion is regulated both by kisspeptin and its receptor kiss-R1 and by neurokinin B and its receptor, whose stimulatory effects increase during puberty; likewise, dynorphin and its receptor, whose inhibitory effect is repressed, are also associated with increased GnRH secretion [10]. Gamma aminobutyric acid (GABA) is the major neurotransmitter associated with the inhibition of GnRH secretion during childhood, whereas glutamate, neuropeptide Y, endorphins, opioids, and melatonin are involved in activating the GnRH pulse generator and hence triggering the onset of puberty [2,10]. In particular, a key role in early pubertal timing is played by leptin and kisspeptin. The action of these two metabolic hormones, in addition to depending on the specific genetic background of the individual [14], is also influenced by epigenetics [15]. The kisspeptin system is influenced by both excess and defective premature malnutrition. Notably, one study also demonstrated that fetal underfeeding affects the production of this hormone, directly impacting the timing of puberty onset in mice [16]. Furthermore, pubertal timing is normalized by the chronic central injection of kisspeptin [17]. The leptin circulation level is correlated with the birth weight and increases significantly just before pubertal onset [2]. It is regarded as a crucial factor in both the timing of puberty onset and caloric balancing, as well as being a major factor in fertility [4]. Recently, it was reported that leptin may also be considered a positive regulator of the kisspeptin system, although the link has not been fully defined [18].

During puberty, two main events occur: gonadarche, which is the activation of the gonads by the pituitary hormones follicle-stimulating hormone (FSH) and luteinizing hormone (LH), and adrenarche, that is, the increased production of androgens by the adrenal cortex [8]. 

The most recognizable physical and biological signs during puberty are statural growth (approximately 20% of adult stature matures during puberty) and the acquisition of secondary sexual characteristics [19]. The most widely employed staging system is that of sexual maturity assessments, also referred to as “Tanner stages”, initially proposed by Marshall and Tanner [20,21]. These are systematized descriptions of the development of secondary sexual characteristics, specifically, the changes in breasts in females (thelarche), genitalia in males, and pubic hair (pubarche) in both sexes. The changes in these three areas are each described in five stages, with stage one representing prepuberty and stage five representing adult development [8]. In addition, several terms are used to describe specific pubertal events: thelarche (the appearance of breast tissue), menarche (the first occurrence of menstrual bleeding), spermarche (the initiation of sperm production), and pubarche (the appearance of pubic hair, but the same term also applies to the first occurrence of axillary hair, apocrine body odor, and acne) [8].

#### 3.1.2. Timing of Puberty

The genetic background is responsible for 50–80% of the variability in pubertal timing [22]. Ethnicity, particularly African American and Hispanic, is classically associated with the earlier onset of puberty. Findings suggest that, regardless of age, Black and Hispanic teenagers precede White adolescents in pubertal development [23]. The earlier onset of puberty in Black youths has been ascribed to genetic factors, higher body mass index (BMI), and nutritional factors. No ethnic variations were observable in the duration from one stage of sexual maturity to the next for girls. In contrast, sexual development in Black and Hispanic boys took longer than in White boys [23].

Childhood obesity may contribute to the decline in the age of puberty onset, and its prevalence has clearly increased over recent decades [23,24,25,26,27,28,29,30,31]. The interaction between hormones and nutrition during fetal life and infancy, as critical periods of growth, is crucial to future pubertal development [2]. A meta-analysis and systematic review by Li et al. [30] revealed that obesity may contribute to early pubertal development. The evidence suggested that obesity and high BMI may promote early pubertal onset in girls, but no statistically significant differences were found in the age of menarche between obese and normal-weight girls [30,32]. For males, not many studies in the published literature have explored this topic, but some authors suggest that a higher BMI corresponds to the earlier development of male genitalia and consequently reduced pubertal timing [30,33].

Exposure to chemicals capable of interfering with the proper functioning of the endocrine system provides another plausible explanation, but further investigation is needed to clarify the effect of these substances [25,34,35,36]. A variety of studies reported in the literature have shown that incidental exposure to sex steroids is associated with early puberty in children. Characteristically, estrogens cause breast development, while androgens cause skin changes (oily skin and hair, adultlike sweat odor) and pubic hair growth. Ointments and salves containing estrogenic ingredients, e.g., lavender oil, have also been associated with prepubertal gynecomastia [37]. Further studies are needed to clarify the role played by these substances, such as phthalates, dioxins, polybrominated biphenyls, and polychlorinated biphenyls, in pubertal timing [22,38,39,40]. However, potential mechanisms of action have been delineated: for example, they can act as hormone agonists or antagonists, or they can alter the levels of endogenous hormones and their ratios by affecting their production, secretion, binding to transporters, metabolism, and excretion [22].

Prenatal factors and specific exposure during fetal life should not be underestimated [25,41,42,43,44]. Maisonet et al. [44] investigated several maternal variables (level of education; social class; age at menarche; prepregnancy BMI; race; age at delivery; season of delivery; previous live births; presence of urine sugar in the third trimester; and smoking and alcohol, coffee, and tea consumption during pregnancy) to identify a potential association with pubertal timing in offspring. These data were collected through self-assessment by the participants of this study. It was found that an onset of menarche before the age of 12 years, smoking during pregnancy, and primiparity were maternal prenatal characteristics associated with earlier pubertal timing [44]. Some studies have shown that accelerated bone maturation and peak height velocity occur at an earlier pubertal stage in small-for-gestational-age (SGA) children than in appropriate-for-gestational-age (AGA) children, resulting in a shorter duration of pubertal growth and lower than expected peak pubertal growth. In addition, it appears that rapid weight gain and visceral adiposity responsible for insulin resistance in early childhood may influence pubertal onset in SGA-born children [45,46,47].

Earlier pubertal timing is associated with the development of several health sequelae, both physical and psychological [48]. Indeed, the timing of pubertal maturation has an impact on self-esteem, behavior, growth, and weight. In addition to physical changes, cognitive and psychosocial maturation also occurs [8,49]. Specifically, there is growing evidence that earlier pubertal timing, particularly a lower age at menarche among girls, is associated with increased risks of obesity, type two diabetes [50], and cardiovascular disease [51,52]. Other reported associations include increased risks of breast cancer [53], lower bone mineralization [54,55], and increased all-cause mortality [56]. Some studies have described negative associations between early puberty in men, in relation to their voices breaking, and adiposity [57], higher blood pressure [58], and cardiovascular disease later in life [51,52].

Additionally, it has been shown that early pubertal timing seems to be associated with psychological and psychiatric disorders; in particular, some scientific evidence classifies it as a predictive factor of substance abuse among adolescents [59]. We can also safely say that early puberty has an association with poor behavioral and psychosocial outcomes and good health in adulthood [49]. In Figure 1, the determinants of the timing of puberty and the complications related to PP are shown.

The normal spectrum of the onset of puberty among children is fairly broad, and the timing varies widely according to gender and ethnicity. 

The age at the onset of puberty has traditionally been considered a reliable indicator of the general population’s well-being [34], and, as a result of changes in society, epidemiological studies have shown that the timing of normal puberty has significantly changed over the last several centuries [9]. Although a lowering of the age of onset of puberty was observed in both sexes, this was undoubtedly more evident in girls compared to boys [24]. Indeed, records for the date of menarche, in both retrospective and prospective studies, were easier to collect. 

As early as 1800, records of menarche age have been recorded and noted in the clinical records of particular groups in the United States and Europe [34]. European historical records indicated a marked drop in the age of menarche from about 17 years in the early 19th century to about 13 years in the mid-20th century [9]. Similarly, declining tendencies occurred in the United States during the first half of the 20th century [9]. Since the 1960s, the age at menarche appears to have stabilized in both Europe and the United States, although minor but still statistically significant reductions of 2.5–4 months have been reported over the past 25 years [9]. In contrast to the onset of menarche, the age of breast development onset seems to have decreased significantly over the past two decades, decreasing from around 11 years before the 1980s to below 10 years of age, according to the Third National Health and Nutrition Examination Survey (NHANES III), conducted in the United States of America from 1988–1994 and based on self-assessment of breast development, and the Pediatric Research in Office Settings (PROS), founded by the American Association of Pediatrics in 1988 and based on breast palpation, in 39% of the cases [9]. Differences in relation to ethnicity are evident; the most pronounced changes were observed in non-Hispanic Black girls. However, a reduction in the age at the onset of breast development was reported in all ethnic groups. Specifically, in the PROS study, at eight years of age, the diagnostic limit for considering PP, 6.7% of White girls and 27.2% of African American girls met the criteria for PP on the basis of breast or pubic hair development [9]. In light of this, the Lawson Wilkins Pediatric Endocrine Society (LWPES) reconsidered the age limit for the assessment of PP, indicating that suspicion of PP is warranted if secondary sexual characteristics appear before seven and six years of age in White and African American girls, respectively [9]. However, other pediatric endocrinologists disagree, as the PROS study was not representative of the general U.S. population [9].

However, according to recent reports by Biro et al. [60], there has been an increase in the rate of Black and White girls presenting with thelarche before the age of eight years (10.4 and 23.4%, respectively) [9]. The Copenhagen Puberty Study reported a 12-month decline in the mean age at the onset of breast development over a 15-year period in Danish girls, and similar data on puberty onset were recently recorded in other European countries [9,61,62]. According to observations from the NHANES III and Third National Health Examination Survey (NHES III) studies, no evidence was found for an advancement in the age at the onset of stage III-V breast development in American girls in general, which indeed, in the Copenhagen Puberty Study, appears to have declined in parallel with the decline in age at the onset of breast development [9]. 

Not as much attention has been paid to male pubertal timing, which is why fewer studies exist and are based on smaller population numbers [9]. Since the earliest reports from the 1940s, the pubertal age of boys evaluated by American and European studies has been consistent, with the onset occurring at approximately 11.5 years of age [9,21,63]. An earlier onset of genital development was evident in NHANES III than was previously reported in US males by NHES III [9,64,65]. However, the interpretation and comparison of the data reported by the NHES III and NHANES III studies are not straightforward, due to the lack of data on pubertal onset in NHES III and the lack of rigorous interobserver policies in NHANES III, which exposes the risk of the misclassification of the onset of puberty in prepubertal boys [9,63,64,66,67]. In addition, a contemporary American study using an orchidometer as a tool to assess Tanner’s G stage observed that the age at the onset of puberty was consistent with previous American and European studies [9,25,64]. Unfortunately, though, the orchidometer has not been used in any of the population-based studies [9]. Nonetheless, the Copenhagen Puberty Study [65] documented a three-month decrease in the age at puberty onset over a recent 15-year period, evaluated by both genital staging and orchidometer. A study conducted by PROS concluded that, only when properly trained, experienced clinicians in this area are able to accurately identify signs of the onset of puberty through Tanner staging [21,68] in boys. The age limit of nine years classically used to define precocious puberty is still valid in boys, and the LWPES found no evidence to support lowering their diagnostic age limit [18], unlike the claims for the female sex [9]. A secular trend analysis between NHES III and NHANES III did not find convincing evidence supporting an earlier age among boys entering genital stages 3–5. In contrast, the Copenhagen Puberty Study found that the onset of later pubertal stages was reached at an earlier age, and the annual increase in testicular volume was greater at the time of the study than 15 years earlier [9,65].

#### 3.1.3. Precocious Puberty

PP is defined as the beginning of pubertal development before eight years of life in girls and nine years of life in boys [5,69,70,71,72]. PP can be divided into Central Precocious Puberty (CPP) and Peripheral Precocious Puberty (PPP). The common causes of CPP and PPP are summarized in Table 1 [70].

The incidence of CPP varies significantly between different cohorts. In American girls, the incidence was estimated at around 1 in 5.000–10.000, while in a Danish study, the prevalence was 1 in 500 [5,69,73]. CPP is more frequent in girls than boys (female/male ratio from 3:1 to 15–20:1) [5,73]. CPP can be due to congenital or acquired central nervous system (CNS) lesions or monogenic defects, or it can be idiopathic [70].

Idiopathic CPP is responsible for most cases of CPP, and it is more frequent in girls than in boys (90% of cases vs. 30–50%). Many different factors have been studied in an attempt to explain idiopathic CPP, especially metabolic and environmental factors [70]. Among the metabolic influences on pubertal timing, it is well known that leptin, an adipocytokine produced by adipocytes, can have a permissive role in the onset of puberty, stimulating kisspeptin release [74]. A more exhaustive analysis of the correlation between nutrition and the timing of puberty is presented in the following paragraph. Metabolic and environmental causes should also be considered in the case of adopted children, who are known to be at higher risk of CPP [70]. Some authors have suggested that this could be explained by nutritional deprivation in early life, followed by increased nutrition after adoption [70]. Others have speculated that environmental factors such as stress and early-life exposure to endocrine-disrupting substances can also have a role in CPP in adopted children [5]. The CNS lesions that are most often involved in causing CPP are listed in Table 1. CNS lesions are far more frequent in boys and young children. In particular, hypothalamic hamartomas are the most common organic cause of CPP [5,73]. Hamartomas usually occur before four years of age, causing seizures or gonadotropin-dependent PP with negative tumor markers for PPP [73]. The monogenic causes of CPP have been a fascinating object of study in the last decade [75,76,77]. Different monogenetic defects have been discovered: the gain-of-function mutation in the kisspeptin excitatory pathway (mutations in genes KISS1R and KISS1) and the loss-of-function mutation in the makorin RING finger 3 (MKRN3) gene and in the imprinted Delta-like 1 homolog (DLK1) gene [69,78,79,80]. Some authors have also studied polymorphisms related to precocious susceptibility [81]. In contrast to CPP, PPP is a gonadotropin-independent process in which sex steroids are produced without hypothalamic and pituitary stimulus. PPP can be caused by the production of sexual hormones by the gonads or adrenal glands, gonadotropin production by tumors, or exposure to exogenous hormones [70,82].

The diagnosis of PP is made on the basis of an accurate personal and familial history, a complete physical examination, and hormonal and radiological exams. It is essential to investigate the parental timing of puberty, the timing of onset of pubertal changes in the child, the possible exposure to sex steroids, and the presence of the symptoms of CNS lesions (visual abnormalities, new-onset headaches, seizures) [5,83].The physical examination should be focused on anthropometric measurements (weight, height, BMI, and growth velocity) and the assessment of secondary sexual characteristics (breast buds in girls, testicular volume in boys, pubic hair in both), according to the Marshall and Tanner classification [20,21].

Other signs of pubertal development are the enlargement of the labia majora and minora, the redistribution of body fat in the hips, and vaginal discharge in girls, whereas voice change and increased muscular mass should be evaluated in boys. The evaluation of axillary hair, oily skin, and facial acne in both sexes should also be considered [5,73,84].

The initial hormonal evaluation entails measuring basal serum gonadotropins and sex steroids. A basal morning LH value of more than 0.2 mUI/mL, measured with an ultrasensitive methodology, is usually considered indicative of puberty [5,70,71,84,85,86]. Moreover, children with early CPP could have a normal basal LH, and children younger than three years of age usually have higher baseline gonadotropin concentrations [5,84,85]. The GnRH stimulation test remains the gold standard [71,87,88]. A peak of LH higher than 3.3–5.0 UI/L is usually diagnostic of CPP [5,89]. In addition, an LH to FSH ratio higher than 0.6 has been associated with CPP [84,85].

Other hormonal evaluations should include thyroid tests, testosterone, estradiol, 17-hydroxyprogesterone (17-OHP), carcinoembryonic antigen (CEA), cancer antigen 125 (CA125), alpha-fetoprotein, and beta-human chorionic gonadotropin (beta-hCG), depending on the patient’s history [73]. 

An advanced bone age of more than 2.5 standard deviations (SD) or more than two years is likely associated with pathological precocious puberty [5,87]. Nevertheless, a normal bone age does not exclude precocious puberty [5,71]. 

In girls, a pelvic ultrasound should be performed in order to assess the premature pubertal development of the ovaries and exclude the presence of ovarian cysts or tumors [90,91]. Although a pelvic ultrasound is a useful tool, some authors do not agree on the diagnostic threshold for the uterine and ovarian volumes [85]. However, a uterine longitudinal diameter ≥3.5–4 cm, a transverse diameter ≥1.5 cm, the presence of an endometrial echo, and an ovarian volume ≥2 cm^2^ have been observed to be associated with rapid progressive CPP [71,84,86].

Brain magnetic resonance imaging (MRI) is suggested in patients with a diagnosis of CPP to rule out CNS lesions [5]. These are more frequent in boys than girls and in young children (before the age of six years) [5]. For these reasons, some authors have suggested that MRI should not be performed routinely in girls between six and eight years of age [85,86,87]. In patients with a clear family history of precocious puberty (two or more family members affected), a genetic analysis is also suggested [5]. CPP should be treated according to the underlying pathology [92,93]. Although surgery and radiation may be indicated in some CNS lesions, the standard treatment for idiopathic and monogenetic CPP is a GnRH analog (GnRHa). GnRHa therapy is clearly indicated for patients presenting with early CPP with advanced pubertal development according to the Tanner stage and with increased linear growth [73,86,94].

The treatment goals of GnRHa are to slow pubertal progression in order to preserve the adult height potential in both boys and girls. This goal is more easily reached if the treatment is started before six years of age in girls [85]. Reducing the health risk and preventing the psychological consequences associated with precocious menarche described in the previous paragraph are also important outcomes in girls [86]. Because these topics have been poorly studied in boys, they are usually less frequently considered when treating CPP in males [92]. The treatment is usually withdrawn to let the puberty of the patient progress concurrently with their peers. Therefore, the treatment is usually discontinued between 10 and 11 years of age for girls and 12 and 13 years of age for boys [95]. Other authors withdraw the therapy at a bone age of around 12 years in girls and 13 years in boys [5,96].

Long-acting GnRH therapy shows good tolerability and efficacy. The most common side effects are headaches, hot flashes, local skin reactions, sterile abscesses, nausea, and vaginal bleeding at the beginning of the treatment [84,87]. Most side effects are transient and do not require therapy discontinuation [93]. Several studies have focused on the long-term outcomes of GnRHa therapy. For example, different studies have shown an increase in adult height in patients treated with GnRHa for CPP [97]. No evidence was found for the possible impairment of reproductive function in either female or male children treated for CPP. Similarly, no clear evidence was found for an increased risk of polycystic ovary syndrome (PCOS) in girls. Moreover, GnRHa does not appear to increase the risk of obesity or reduced bone mineral density during adulthood in either boys or girls [84,85,86,92,93,98,99].

### 3.2. Diet and Timing of Puberty

#### 3.2.1. Maternal Nutrition

An important issue to consider is the relationship between maternal status (in terms of BMI and nutrition) during pregnancy and the eventual development of PP in offspring. 

Different studies have examined the relationship between maternal adiposity and the daughter’s menarcheal age. However, the findings are discordant: some authors reported inverse linear relationships between the maternal BMI [100,101] and the daughter’s age at menarche, while others did not find any associations between the two parameters [56,102]. On the other hand, recently, the Avon Longitudinal Study of Parents and Children [103], a prospective population-based cohort study, demonstrated that both a high prepregnancy BMI and a greater gestational weight gain (GWG) are associated with earlier puberty in daughters. This association was mediated by a higher prepubertal BMI in the daughters [103].

In this context, nutrition has multiple roles. Firstly, an excessive caloric intake during pregnancy leads to an excessive GWG [104], and a high GWG is considered one of the main contributors to childhood overweight development [105,106] and its consequences in terms of puberty [107]. Moreover, consuming a high-fat diet during pregnancy was found to contribute to gut dysbiosis and an increased risk of obesity in the offspring [108,109,110] that predisposes to PP development [107]. In addition, the dietary intake of specific bioactive compounds such as phytoestrogens during pregnancy was thought to influence puberty development in children [111]. Phytoestrogens, such as genistein, daidzein, O-desmethylangolensin (O-DMA), enterodiol, and enterolactone, are natural estrogenic compounds [112]. Two important groups of phytoestrogens are isoflavones (found in legumes, especially in soy) and lignans (found in seeds, cereal grains, and some fruits) [113]. Exposure to phytoestrogens is mostly dietary and these elements can cross the placental barrier [114,115]. Studies in rodents found that isoflavones during gestation can lead to early vaginal opening (mimicking early menarche in humans), irregular cyclicity, and decreased GnRH activation [116,117]. Recently, Marks et al. investigated the association between in utero exposure to phytoestrogens and early menarche, examining data from the Avon Longitudinal Study of Parents and Children, and observed a positive association between the maternal enterodiol and O-DMA urine levels and the decreased risk of early menarche in the offspring [112]. Nevertheless, further human studies concerning this topic are needed for conclusions to be made.

#### 3.2.2. Early-Life Nutrition

Nutrition is considered one of the most important factors affecting pubertal development [6]. In terms of pubertal onset, an important link between nutrition and puberty lies in the relation between overfeeding and the consequent weight gain and obesity development [107]. Indeed, a low birth weight, followed by rapid weight gain and high body weight in infancy and childhood, has been correlated with an early onset of puberty and early menarche [2,102,118,119,120]. It is important to underline that nutrition can influence pubertal timing independently of weight gain, for instance, acting on infant–parent attachment [121,122,123]. 

When discussing early-life nutrition (before 2 years), two main topics should be considered: breast or formula feeding and the transition to a complementary diet.

##### Breastfeeding

Breast milk is the first food that newborns should ideally consume; indeed, the World Health Organization recommends exclusive breastfeeding from birth to six months of life [124]. The American Academy of Pediatrics also recommends breastfeeding for at least 12 months [125]. Moreover, the Academy of Nutrition and Dietetics highlights that exclusive breastfeeding provides optimal nutrition and health protection in the first six months of life, and the addition of complementary food from six to twelve months of age is the ideal feeding pattern for infants [126].

Different studies have attempted to evaluate the influence of breastfeeding on the process of pubertal development, but the results are not always coherent. Two European cohort studies examining the relationship between breastfeeding and early puberty onset did not find an association between breastfeeding and menarche age [119,127]. Breastfeeding was not associated with pubertal age in non-Western settings either, suggesting that the correlation may vary by context [128]. In a recent population-based cohort study in 13,511 boys and girls, a shorter duration of breastfeeding was associated with earlier pubertal development in boys but not in girls. Specifically, boys who were never breast-fed attained pubertal markers earlier (~4.1 months) than boys exclusively breastfed for at least four months [129]. In girls, the duration of breastfeeding was not associated with pubertal development [129].

On the contrary, breastfeeding was shown to postpone sexual maturation in girls at risk of early puberty onset: specifically, Aghaee and colleagues observed that non-breastfed girls were more likely to experience earlier thelarche compared to girls breastfed for six months. These results were obtained by evaluating 3331 mother–daughter pairs and adjusting for ethnicity, maternal age, and education level [121]. Furthermore, the Cebu Longitudinal Health and Nutrition Survey evidenced a 6% decrease in the risk of earlier menarche for every one-month increase in exclusive breastfeeding [130]. Moreover, evaluating 219 Korean children, Lee et al. found that children breastfed for more than six months had a lower risk of early pubertal development (in terms of Tanner stage ≥ 2 at nine years) compared to children breastfed for less than six months [131]. In a UK retrospective study, Morris et al. observed delayed menarche in women who were breastfed as infants [132]. Therefore, the evidence seems to support the protective role of breastfeeding against early puberty onset. 

An important link between nutrition, including early-life feeding, and pubertal onset lies in the relation between overfeeding and the consequent weight gain [107]. Overfeeding in infancy may in fact result in overweight development and earlier puberty [102,118,120,133]. Interestingly, breastfeeding during the first year of life has been shown to decrease the odds of childhood overweight incidence by 15% when compared with formula feeding [134]. The protective effect of breast milk in terms of weight gain lies in the difference in growth rates between breastfed and formula-fed babies [102,135,136]: infants fed with breast milk show a slower growth curve relative to formula-fed children, and this seems to have a protective role against obesity development later in life [136,137,138]. The association between rapid weight gain, the so-called ‘catch up growth’, and obesity was shown to be significant, even after adjusting for infant birth weight [139].

Different factors are thought to interact in the relationship between breast milk and prevention of both overweight and precocious puberty, such as insulinlike growth factor (IGF)-1 [120,140] and leptin [141,142] levels, different bioactive nutrients present in human and formula milk [136], the microbioma composition [6,143], and appropriate self-regulation of feeding [136,144]. Increased levels of IGF-1 were indeed detected both in formula-fed infants [140] and in babies who experienced fast weight gain in the first months of life [145]. Importantly, high IGF-1 levels have been correlated with enhanced sex steroid production [146] (facilitating GnRH secretion [147]) and pubertal development [148]. 

It is also worth mentioning that, besides weight gain, some psychosocial factors may be involved in the association between early-life feeding and pubertal onset, such as early childhood experiences: indeed, infant–parent attachment, which was found to positively correlate with breastfeeding [149], seems to regulate the reproductive development later in life [122], and maternal depression, which was observed to inversely correlate with breastfeeding [150], has been linked to increased risk of earlier pubertal timing in girls [151].

##### Formula Feeding

Infant formulas are considered effective substitutes for breast milk and are created to mimic the nutritional composition of human milk [152]. There are three major classes of formulas: cow-milk-based formula, soy-based formula, and specialized formula [152].

When discussing differences between breast and formula milk, it is fundamental to analyze the nutrient composition: the majority of formula milk formulations are higher in energy and protein and lower in fat compared to breast milk [136]. Specifically, formulas have been estimated to contain as much as 50–80% more protein compared to breast milk, and the higher protein content has been considered the main culprit in the differences in weight between breastfed and formula-fed infants [135,153,154] and, importantly, in terms of obesity development [155,156]. In particular, the protein content in human milk ranges from 1.4–1.6 g/100 mL (in early lactation), to 0.8–1.0 g/100 mL (after three to four months of lactation), to 0.7–0.8 g/100 mL (after six months) [152], whereas infant formulas have a protein content from 2–2.5 g/100 mL to 2.9 g/100 mL (formulas with a higher protein content are mainly used for very low birth weight or preterm infants) [152,157]. 

According to the early protein hypothesis, the higher protein content in infant formulas could lead to increased circulating levels of IGF-1, resulting in accelerated growth and greater adiposity [158]. Besides different nutrient substrates, formula milk is more energy-dense, and this may have an obesogenic effect [159]. Interestingly, an obesogenic effect was also correlated with bottle use: feeding from a bottle leads to worse self-regulation of satiety relative to breastfeeding, increasing the risk of overweight development [144]. Novotny et al. analyzed the age, ethnicity, menstrual status, and feeding pattern during infancy among 340 girls from nine to fourteen years old and observed increased body fat deposition, with consequent earlier attainment of menarche, in formula-fed girls compared to those who were breastfed [160].

An additional factor to consider when discussing early-life nutrition is gut microbiome development: indeed, breast milk is considered a significant factor involved in the microbiome structure of infants [161,162], and infants fed with human and artificial milk seem to have different microbiome compositions. Formula feeding has been associated with a less stable microbiota, characterized by a different bacterial composition and a higher bacterial diversity compared with breastfeeding [162]. Emerging evidence suggests that the gut microbiome, specifically the proportion of Firmicutes- to Bacteroides-type bacteria, has a relevant role in obesity development [163,164,165]. However, few data exist to date, and further studies are thus needed to clarify this association and draw conclusions concerning this topic and the possible repercussions on pubertal onset.

##### Soy-Based Formulas

When discussing infant nutrition and pubertal timing, it is also worth mentioning soy-based feeding. Formulas made from soy protein are useful options for infants with galactosemia or congenital lactase deficiency and are also used for infants with milk allergies [152]. Because of the phytoestrogen content in this type of formula, its use is limited by the concern of potential harm to infants [152,166], but evidence on this subject is controversial [167].

Agdent et al. analyzed the timing of menarche in relation to infant feeding methods in 2920 girls, evaluating the potential effects of soy isoflavone exposure through soy-based infant feeding. They reported a small increased risk of menarche in early adolescence in soy-fed girls compared to their non-soy-fed peers [167]. Specifically, early soy-fed girls were at 25% higher risk of menarche during the follow-up compared to girls fed with non-soy-based infant formula or milk [167]. These findings may be due to the weak estrogenic effects of some soy isoflavones, specifically genistein and daidzein, present in various soy products [167].

Evidence of estrogen exposure through soy was also detected in the urogenital epithelium and uterus of infants fed with soy formula [168]. Furthermore, an association was recently suggested between manganese, present in higher amounts in soy formulas, and early pubertal onset [169,170]. In addition, in a retrospective case-control study analyzing 161 girls, 84 of whom were affected by central precocious puberty, Felìcio et al. found that the use of soy was associated with central precocious puberty, whereas exclusive breastfeeding was a protective factor [171]. Specifically, exclusive breastfeeding for more than six months was shown to be negatively correlated with the presence of the pathology (*r* = −0.2; *p* < 0.05), whereas the use of soy was shown to be significantly higher in the group of girls affected by central precocious puberty (OR: 3.8; IC 95%: 1.5–6, *p* < 0.05) and positively correlated with the presence of the disease (*r* = 0.2; *p* < 0.01) [171]. Moreover, the duration of the soy intake (in terms of years) was correlated with the bone age [171]. On the other hand, Giampietro et al. evaluated the hormonal and metabolic effects of long-term (more than six months) soy-protein formula feeding in 48 children and did not find any correlation between soy feeding in early life and hormonal effects: neither signs/symptoms of precocious puberty in girls nor those of gynecomastia in boys [172]. The bone age was also within the normal range [172]. In a nested case–control study prospectively examining the possible association between soy-formula feeding and early pubertal signs, Sinai et al. found no difference in pubertal development, growth, or BMI values among those provided with soy-based formula during infancy [173].

Thus, although the association between early soy exposure and early menarche is biologically plausible, different studies have produced conflicting results [167,168,172] and further research is needed to elucidate this topic.

##### Complementary Feeding

Another important factor to consider in the context of early-life nutrition, especially from six months of life, is the transition from breast/formula feeding to a complementary diet. Interestingly, both the timing [174] and the type [175,176] of complementary solid food introduction seem to be associated with an increased risk of childhood obesity [137,177] and, possibly, with precocious puberty [2].

The late introduction of complementary feeding was shown to be protective against overweight later in life [178], and the introduction of solid foods before four months was associated with a six-fold increased risk of obesity development at three years in formula-fed babies [174]. Thompson and Bentley examined the infant characteristics associated with age-inappropriate feeding among mothers and children participating in the U.S. Infant Care and Risk of Obesity Study (a cohort study of 217 low-income, first-time mothers and infants followed from three to eighteen months of age) and found that more than 75% of infants received solids or juice by three months of age [175]. Inappropriate feeding was also associated with a higher daily energy intake and an increased weight-for-length, factors known to increase the long-term obesity risk [175]. In terms of specific nutrients and overweight risk, a high polyunsaturated fat intake was associated with a lower risk of overweight [175], whereas a high protein consumption was associated with a higher risk of adiposity development [176,179]. Analyzing children of the Danish National Birth Cohort, Morgen et al. examined whether protein intake at the age of 18 months and the timing of the introduction of complementary food could be associated with the body mass index and overweight at ages seven and eleven years. They found that an earlier introduction of complementary food (before four months) was not associated with an increased BMI at age seven years, but it was associated with a higher BMI at eleven years. Moreover, protein intake from dairy products was associated with increased BMI values at age seven, and protein intake from meat and fish was linked to a higher BMI (both at seven years and at eleven years) and increased odds of overweight at age seven [179]. As with milk, complementary feeding also influences the gut microbiota and the consequent risk of later obesity [164]. Unfortunately, studies on the relationship between complementary feeding, intestinal flora, and early puberty are lacking.

An additional issue to consider when discussing complementary food and puberty is the introduction of soy-based foods. Indeed, this issue has been extensively researched, primarily because of the amount of isoflavones provided by these foods [166,180]. Isoflavones, initially classified as phytoestrogens, have been more recently considered selective estrogen receptors [180]. In their review, Chakraborty et al. [167] suggested that phytoestrogens may play a role in precocious puberty [181]. Unfortunately, little soy-related research involving children has been conducted, but it was shown that, in Asia, soy is consumed at a very early stage of life (between six and twelve months of age), mainly as tofu and miso soup [180,182]. Consumption of soy-based foods at early ages was also reported by Wada et al. in a survey involving more than 400 Japanese boys and girls aged three to six years [183]. Moreover, it was reported that, in Singapore, 70% of healthy children younger than ten years consumed soy products. Of these, >95% consumed this type of food prior to eighteen months of age [184]. Unfortunately, although there is a particular interest in understanding the effects of isoflavones in young people, there has been limited investigation of the effects of soy-based foods on pubertal onset, especially for evaluating the introduction of these foods at an early age [180]. Two Korean case–control studies demonstrated higher levels of urinary isoflavones in girls with precocious puberty relative to the controls [185,186]. Indeed, Kim et al. showed that girls (aged 8.6 ± 0.8 years) with central precocious puberty had significantly higher serum concentrations of total isoflavones compared to the age-matched controls (77.9 ± 57.2 nmol/L vs 62.9 ± 40.2 nmol/L) [185]. Yum et al. identified a higher plasma genistein concentration in girls with precocious puberty (aged 8.91 ± 1.40 years) compared to the control peers (8.12 ± 12.71 ng/mL vs 3.04 ± 4.21 ng/mL) [186]. In agreement, Segovia-Siapto et al., evaluating 248 boys in a cross-sectional study, found that pubarche manifested earlier in those who consumed moderate/high amounts of soy compared to the low-soy consumers [187]. On the other hand, a prospective study involving 1239 girls aged six to eight years living in the United States did not show any relation between pubertal development and urinary isoflavone excretion [188]. Moreover, Cheng et al. examined whether the intake of isoflavones in healthy children before their pubertal growth spurt could be associated with puberty timing and showed that girls with higher prepubertal isoflavone intake appeared to enter puberty at a later age [189].

As reported in the literature, only a few studies involving children have been conducted, and data are limited; thus, more research concerning this fascinating issue is needed for conclusions to be drawn.

**Figure 2:**
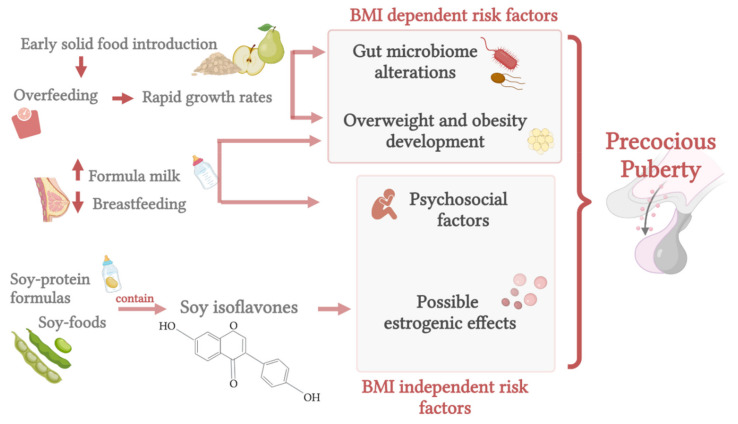
Early-life nutrition and the possible effects on precocious puberty (created with BioRender.com (accessed on 9 November 2021)).

The influences of early-life nutrition are summarized in Figure 2.

#### 3.2.3. Childhood Nutrition

Considering childhood nutrition (between 2 and 12 years), a healthy diet should ensure a balanced energy intake and an adequate supply of macro/micronutrients. The energy intake should be determined individually according to individual needs, energy expenditure, and growth. A commentary by the ESPGHAN Committee on Nutrition recommends eating at least four meals a day, with a strong emphasis on breakfast. The food portions should be appropriate for the age and nutritional status. Snacks should be healthy, and the consumption of energy-dense foods should be avoided. The intake of fast-absorbing carbohydrates and simple sugars should be limited in favor of slow-absorbing carbohydrates. Sugar-sweetened drinks should be avoided, and daily water intake should be ensured. The fat intake should meet, but not exceed, the nutritional requirements, and polyunsaturated fatty acids (PUFAs) should be preferred. The high consumption of plant-based foods should be ensured with adequate monitoring of the nutrient intake [190]. Several nutrient-based studies suggest that childhood diet is a modifiable risk factor for precocious puberty. A combination of different nutritional aspects, rather than a single dietary factor, influences the time of sexual maturation [191]. Adherence to specific nutrient recommendations at the pediatric age results in a higher dietary quality and is relevant to the timing of puberty. 

##### Energy Imbalance: High-Energy Diet and Body Composition

A high-energy diet and the resulting increased prepubertal BMI are associated with an earlier onset of puberty. Nguyen et al. [192] reported a significant correlation between a higher energy intake during childhood and PP in girls with elevated BMI levels. This novel result is important, as previous studies have not suggested a consistent association between childhood energy intake and the onset of puberty and have not clarified whether body composition has a greater impact on the onset or duration of puberty [123,193,194,195,196]. Evidence from studies of female mice and monkeys has also shown that a high-energy diet and the subsequent gain in body fat cause increased levels of leptin, a signal to the brain that leads to the onset of puberty [197,198].

If an energy imbalance and an elevated BMI are related to early puberty, a weight reduction can be expected to delay puberty compared to a permanently overweight state or further weight gain. A one-year lifestyle intervention study in overweight/obese children showed a significant reduction in precocious puberty in girls who achieved an improvement in body composition [199]. In prepubertal male rat models, nutritional restriction delayed the reproductive development and reduced the energy imbalance. Short-term refeeding, although with few effects on the body composition, restored the reproductive endpoints (testicular volume and sperm production) and the plasma hormone concentrations (plasma levels of IGF-1, leptin, and insulin) [200].

The relationship between body fat and the reproductive axis in girls is well known and can be regarded as an evolutionary adaptation that ensures pregnant mammals have adequate fat reserves to support both the mother and the growing fetus [28].

However, a small number of reports show a correlation between body fat and PP among boys. Although preliminary longitudinal studies including only boys confirmed an association between a higher prepubertal body mass and early puberty [201,202], a subsequent prospective analysis showed a correlation with a later onset of puberty [33]. Although most studies have reported unequivocal correlations in both sexes, it cannot be excluded that the influence of prepubertal body composition on the timing of puberty may be different for boys [123].

The possible relationship between body fat and early puberty has been the subject of many studies. Leptin is an adipokine secreted by adipose tissue and is considered a permissive factor in the regulation of the HPG axis. The rationale is that there are sufficient energy reserves in adipose tissue to initiate fertility, which is a necessary but not sufficient prerequisite [29]. Leptin concentrations increase before the onset of puberty in girls, and a peak in leptin concentrations precedes a peak in gonadotropin concentrations [203]. An association between the leptin concentration and the timing of menarche has been widely demonstrated, and the resulting increased body fat has been associated with an earlier age at menarche. Thus, leptin appears to be the link between energy imbalance and the HPG axis [29,199].

PP in children with an altered body composition may also depend on peripheral mechanisms: IGF-1 activation, adrenal androgen overproduction typical of obesity, and increased conversion of androgens to estrogens due to the aromatic action of adipose tissue [204,205,206].

A study in mice showed an interaction between insulin and leptin signaling during the peripubertal period [207]. Insulin resistance results in lower concentrations of sex hormone-binding globulin and consequently increases the bioavailability of sex steroids [9]. This results in an earlier onset and an altered rhythm of puberty in obese children with insulin resistance [199].

Leptin levels and adiposity appear to play a direct role in early puberty and an indirect role in cardiometabolic risk in adolescents [208]. Therefore, the regulation of energy imbalance and weight should be recommended to control the timing of puberty and prevent altered cardiometabolic status in pubertal adolescents.

#### 3.2.4. Macronutrients

##### High Protein Intake

Dietary protein intake during childhood is crucial for the timing of puberty, as it promotes the rebound of adiposity before the pubertal onset [2,209]. Furthermore, a high intake of animal protein has been shown to stimulate the HPG axis through the secretion of IGF-1 [192,210].

A prepubertal diet high in animal protein leads to earlier pubertal development. Günther et al. [211] observed that children who consumed a diet with animal protein in the highest tertile resulted in an earlier age (0.6 years earlier) at the pubertal spurt, growth velocity, and menarche compared to boys and girls whose consumption was in the lowest tertile. Similarly, Berkey et al. [212] demonstrated an earlier menarche (0.6 years earlier) among girls with a high intake (8 g per day) of animal protein. 

Although a high intake of animal protein increases infantile adrenal androgen secretion and results in the earlier onset of puberty, Remer et al. [213] showed that these two variables were independent of each other. 

In particular, one study analyzed data from the National Health and Nutrition Examination Survey and showed that a higher milk consumption at 5–12 years of age was associated with an earlier age of menarche [214]. Furthermore, according to the Avon Longitudinal Study of Parents and Children, a higher prepubertal meat intake in a large sample of girls correlated with higher odds of menarche at age 12.5 years compared to their counterparts who consumed less meat [215]. 

However, the dietary intake of milk/dairy products and meat provides key nutrients for the growth and development of children. Therefore, it is imperative to recommend the balanced consumption of dairy products to ensure the intake of essential micronutrients such as calcium, magnesium, and iodine and the balanced consumption of meat to support the intake of zinc, iron, and vitamin B12 [2,192]. 

##### High Fat Intake

Several observational studies have evaluated whether fat intake contributes to early pubertal development because of its potential influence on estrogen metabolism. Although early results provided conflicting data, more recent studies support the hypothesis that a high-fat diet promotes the onset of PP independent of BMI [2].

PUFAs are essential fatty acids involved in the onset of puberty through direct effects on steroidogenesis and mammary gland development [192,216]. Previous studies have shown that a higher PUFA intake at ages 3 and 7 years promoted early menarche [217]. Nguyen et al. [192] demonstrated that a high intake of PUFAs during late childhood was associated with the risk of early menarche, with a dose-dependent effect.

Several studies have investigated the unique effects of omega-3 and omega-6 fatty acids on the onset of early puberty. Preliminary studies showed that a higher intake of omega-3 fatty acids was related to an earlier menarche [218]. Later studies showed that high levels of plasma dihomo-γ-linolenic acid, an intermediate metabolite of n-6 polyunsaturated fatty acids, contributed to promoting early puberty in girls [219]. However, the predominant of the two fatty acids and their optimal ratio to promote a healthy puberty course are still under investigation [123].

The data regarding monounsaturated fatty acids (MUFAs) are conflicting. A preliminary study showed that a high amount of MUFAs could promote the early onset of puberty through the stimulation of mammary gland development and the elevation of serum IGF-1 levels [220]. However, Nguyen et al. [192] demonstrated that a high intake of MUFAs delayed the onset of menarche.

Animal and molecular studies suggest that a high-fat diet induces low-grade hypothalamic inflammation and the subsequent premature activation of GnRH. The potential mechanisms reported are direct microglial activation, the secretion of prostaglandins, neurotrophic factors, and the stimulation of GnRH neurons [221].

A study in mice showed that a high-fat diet led to early puberty with irreversible neuronal damage. Therefore, this study suggested the importance of avoiding high fat intake in childhood to prevent premature puberty and neurodevelopmental damage in mice [222].

Thus, in summary, these findings suggest that a high dietary fat intake may promote the early onset of menarche. However, studies examining male puberty are lacking, and the role of specific fatty acids needs clarification in further studies.

##### High Carbohydrate Intake

Although high intake of sugary beverages and sweets in childhood is known to have adverse effects on children’s health, the effects on the onset of puberty are still unclear. On the topic of the short- and long-term complications, a recent position paper from the European Academy of Pediatrics and the European Group on Childhood Obesity stated that displaying sugary drinks to children and adolescents should be banned [223]. A prospective study found that a higher prepubertal consumption of sugary drinks predicted an earlier menarche onset, regardless of the baseline BMI. Among the sugary drinks tested, the consumption of diet soda and fruit juice showed no such association [224]. Studies have found that a more frequent consumption of sweetened and artificially sweetened soft drinks is predictive of an earlier menarche due to an increase in the BMI, an immediate increase in the circulating insulin concentrations, and the upregulation of hormones [191,225]. Valsamakis et al. [221] also demonstrated that a high-glycemic-index diet causes early puberty through a mechanism of hypothalamic inflammation. However, further studies are still needed to confirm these preliminary hypotheses. Future research could evaluate the effect on puberty in males and on other markers of puberty.

##### Micronutrients

Evidence suggests that the requirements of micronutrients such as iron, zinc, calcium, and vitamin D increase during the pubertal spurt [6]. Therefore, several studies have been conducted to highlight the possible influences of micronutrients on the timing of puberty, but to date, the results have been controversial. Several studies report emerging evidence regarding the possible predictive role of iron, magnesium, zinc, and carotene in early menarche.

A longitudinal study conducted on girls from the Bogota school children cohort (BoSCCo) showed that a higher plasma ferritin status and a greater iron accumulation during childhood were precursors of the early onset of menarche [226]. Subsequently, Nguyen et al. [192] consistently found that a high iron intake was associated with the early onset of menses in girls.

A study found that a prepubertal diet rich in zinc and magnesium was a predictor of early menarche among British girls [217].

A higher carotene intake during childhood has been associated with the onset of early menarche [192]. Although the antiestrogenic effect of carotene is known, a very high intake has the opposite effect, which is exerted in a dose-dependent manner [192,227,228].

Although several studies have investigated the role of fat-soluble vitamins in early puberty, the data are still conflicting and warrant further research. Vitamin D receptors are known to be present in the ovaries, uterus, placenta, testes, and pituitary glands; however, to date, there are no solid data in the literature supporting the role of vitamin D in early puberty [2,229].

Although previous studies have shown that a low childhood intake of vitamin C elevates leptin concentrations, resulting in an early pubertal onset, it was subsequently demonstrated that lower vitamin C levels were correlated with a later menarche age [123,230].

Similarly, conflicting data could not confirm the role of vitamin A intake in pubertal onset [123].

Finally, conflicting results showed that the prepubertal intake of dietary fiber and isoflavones delays the pubertal onset. It has been proposed that the dietary fiber intake affects pubertal development by reducing the availability of circulating estrogen levels [123]. Preliminary studies associated a higher cereal fiber intake at age 10 years with a later onset of thelarche and menarche [231]. However, Cheng et al. [189] showed that fiber intake was not correlated with any pubertal markers (Tanner stage 2 for breast development/testicle volume and age at menarche/change in voice) and pubertal growth in either girls or boys.

Nguyen et al. instead reported that girls with a high intake of fiber and monosaturated fatty acids in childhood experienced a later menarche onset (RR = 0.83, 95% CI = 0.69–1.00, I2 = 31%; RR = 0.66, 95% CI = 0.50–0.86, I2 = 0%, respectively). Thus, an high intake of fiber-rich foods may protect girls from an early menarche onset [192]. Moreover, in a cross-sectional survey undertaken in 1,340 children and adolescents aged 9–15 years, Tian et al. evaluated fiber consumption in relation to the different stages of pubertal development (using Tanner criteria) and reported that the dietary fiber intake, especially fruit fiber, was lower in the children and adolescents with an early commencement of puberty development [232]. Regardless, further studies are necessary to better establish the relationship between dietary fiber and pubertal development.

Isoflavones are nutrients that are structurally and functionally similar to endogenous estrogens and have an inhibitory effect on aromatase [123]. Cheng et al. [189] reported that a high isoflavone intake predicted a delayed thelarche (0.7 years later) and a later pubertal spurt (0.6 years later) in girls. However, dietary isoflavone intake was not implicated in the timing of puberty in boys.

The role of micronutrients in the onset of early puberty is a much-debated topic that lacks solid evidence. Further studies are needed to identify the possible predictive role in male puberty and the mechanisms underlying the associations with the timing of puberty.

#### 3.2.5. Dietary Patterns

##### Mediterranean Diet

The cornerstones of the Mediterranean Diet (MD) are the daily intake of vegetables, fruits, and preferably whole grains. Milk and low-fat derivatives should be moderately consumed, and olive oil should be consumed as a source of fat. Fish and legumes should be moderately consumed at the expense of red meat and eggs. The intake of sweets, soft drinks, and packaged foods should be sporadic, and water should be the main source of hydration [233,234].

This cultural dietary pattern is associated with a reduced weight and a lower abdominal adiposity, with a beneficial effect on anthropometric parameters and cardiometabolic risk factors [235].

Several studies have analyzed the correlation between PP and the macro/micronutrients of this dietary pattern, such as long-chain fatty acids (LCFAs), fiber, vegetables, and red meat [189,192].

Only one study specifically focused on the relationship between MD and the onset of puberty in the pediatric population. Szamreta et al. [236] demonstrated that close adherence to the MD could decrease the risk of PP in a 9–10-year-old cohort of girls in New Jersey. In particular, a significantly reduced likelihood of breast development and a later age of menarche were observed compared to girls with poor MD adherence. The results were independent of the BMI values, body composition measures, or fat distribution. The data analysis suggested that the results were related to a high consumption of fish, vegetables, and low-fat milk.

Further studies are required to confirm this preliminary result and to clarify the mechanism whereby the MD may influence puberty.

##### Vegetarian Diet

The most common type of vegetarianism is the lacto-ovo-vegetarian diet, which includes milk and eggs but not meat or fish. Veganism is a strict vegetarian diet that excludes all foods of animal origin. The risk of insufficient nutrient intake increases with the degree of dietary restriction and poses a risk of malnutrition if micronutrients are not properly supplemented [237].

Although the components of these dietary patterns have been analyzed in relation to early puberty, there is limited evidence correlating vegetarian diets with the onset of puberty.

A previous study showed that a vegetarian lifestyle delays the onset of menarche among well-nourished girls. A high consumption of legumes correlated with an onset of menarche five to six months later. Higher intakes of carbohydrates, thiamine, and iron were associated with the onset of menarche seven to eight months later. In contrast, a higher intake of meat promoted the onset of menarche six months earlier [238,239].

Perksy et al. [240] examined the onset of menarche among vegetarian and non-vegetarian adolescent girls. The diets of the two groups were similar in energy content, but the vegetarian diet was significantly lower in total fat, saturated fat, and protein. The vegetarian girls also had higher intakes of starch and fiber and lower intakes of sucrose and caffeine. This study showed that in the two groups with different diets but an apparently similar energy balance, no differences in the age at menarche were observed (mean age at menarche of both groups: 12.4 ± 1.3 years).

In a study of 481 girls, including 325 non-vegetarian and 156 vegetarians, only 13% of the vegetarians had an early menarche, and 28% had a late menarche. Data on their height and weight were not provided, so this study could not define whether this difference depended on the vegetarian diet influence or the differences in energy balance [241].

Subsequent studies showed significantly lower leptin levels in children on a vegetarian diet compared with children on an omnivorous diet. These findings would support the hypothesis that vegetarianism may delay bone growth and development in childhood [242]. In addition, lower leptin levels in vegetarian children reflect lower body fat stores [243].

There is currently no clear evidence on how the vegetarian diet affects puberty onset. The available preliminary data suggest that, when energy and macro/micronutrients are freely available through the food intake, the age at menarche does not differ between vegetarians and non-vegetarians.

##### New Frontiers in Dietary Patterns

Correlations between PP and new dietary patterns have recently emerged. In the ’lower diet quality’ dietary pattern, total fat and saturated fat predominated at the expense of water, carbohydrates, fiber, vitamin C, folate, vitamin E, sodium, calcium, iron, and a lower energy balance ratio in prepuberty. This dietary pattern was associated with an early puberty (0.4 years earlier) among prepubertal children, regardless of body composition [244].

Chen et al. investigated the effects of three types of dietary patterns, identified as the ’traditional diet’, the ‘unhealthy diet’, and the ’protein diet’, on early puberty among 6–12-year-old schoolchildren in China. The ‘traditional diet’ consisted of vegetables, fruit, red meat, white meat, and seafood. The ’unhealthy diet’ showed a prevalence of sweets, snacks, soft drinks, and fried food. A high load of neogala, protein powder, and dairy products represented the ’protein diet’. Among these, only the ’unhealthy diet’ showed a statistically significant relationship with early puberty in males and females [191].

Duan et al. identified a modern dietary pattern with a high intake of fast foods, milk, fruit, and eggs and a low intake of wheat and vegetables. A high adherence to this dietary pattern during childhood was associated with an increased risk of an early menarche among Chinese girls. The hypothesized mechanisms were based on high milk consumption, high fat consumption, and low plant consumption [245].

Although nutritional research has reported new dietary patterns that promote early puberty, further studies are needed to identify a common dietary pattern that combines macro/micronutrients with a positive impact on the early onset of puberty.

**Figure 3:**
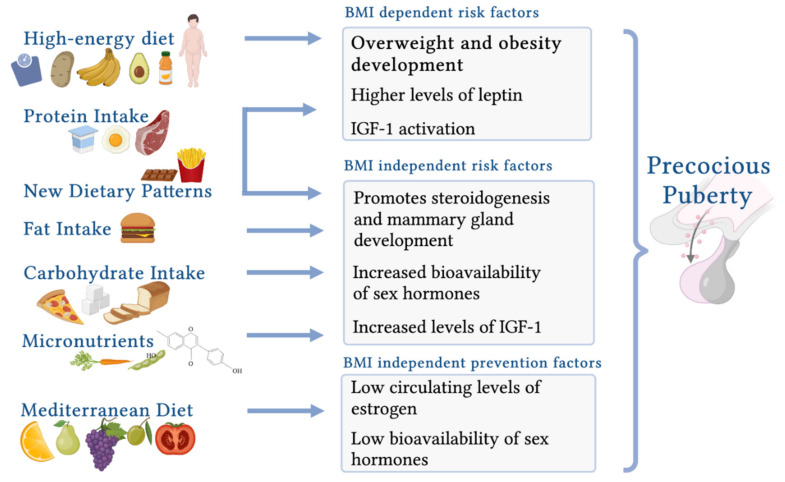
Childhood nutrition and the possible effects on precocious puberty (created with BioRender.com (accessed on 9 November 2021)).

The influences of early-life nutrition are summarized Figure 3.

In Table 2, the role of the diet in early life and during childhood on the timing of puberty is reviewed.

## 4. Conclusions

The timing of puberty represents an important public health issue with clinical and social implications. Even though a major limitation of pubertal timing studies is that they are conducted cross-sectionally, so the possible changes in pubertal progression should be interpreted with caution before being confirmed by longitudinal studies, the literature supports the role of nutritional status and nutrients as determinants of the timing of sexual development.

More than 20% of the variation in pubertal timing may be explained by the nutritional status during early life and childhood. This correlation can be related to rapid weight gain during infancy and childhood, but some effects are also independent of weight gain.

Breast milk, the recommended form of nutrition from birth to six months, seems to have an important protective role against early puberty onset, mainly due to its positive influence on infant growth rate and childhood overweight prevention.

A diet characterized by a high energy, fat, and protein intake and a high glycemic index associated with unbalanced micronutrient supplies seems to be involved in hormonal stimulation, leading to the precocious activation of puberty. Although a high BMI influences puberty timing, unbalanced intakes of fat, protein, and carbohydrates can influence puberty, regardless of body composition. Future studies are needed to clarify the possible preventive and therapeutic roles of micronutrients. The role of the MD and the vegetarian lifestyle deserves further study to assess the correlation with puberty timing. Nowadays, a healthy diet and lifestyle can be proposed as preventive measures for PP. Future research should define a healthy dietary pattern incorporating macro/micronutrients with a beneficial effect on the timing of puberty.

Research on the nutritional determinants of the timing of puberty in boys is relatively scarce. A different effect of the environment on the timing of puberty between the sexes could be supported; further research involving boys is desirable to close this gap.

As a narrative review, this manuscript takes a less formal methodologic approach than systematic reviews. The impact of the study design on the results could be considered; it is well recognized that some research designs are more powerful than others in their ability to answer research questions on the association between risk factors.

However, this study presents an overview on the role of nutritional factors as a modifiable factor for precocious puberty, which is useful as confirmation that it is important to have better knowledge of the mechanism whereby nutrients may influence the regular timing of puberty in order to prevent PP and related complications.

## Figures and Tables

**Table 1:** Causes of precocious puberty.

Central True Precocious Puberty	Pseudo or Peripheral Precocious Puberty	Normal Variant
Idiopathic	Gonadal Ovarian tumor/ovarian cyst Leyding cell tumor McCune Albright Syndrome Familiar testotoxicosis (activating mutation of LH receptor)	Premature thelarche
Congenital central nervous system (CNS) lesion Hypothalamic hamartoma Suprasellar arachnoid cysts Neurofibromatosis type 1 Hydrocephalus Tuberous sclerosis Sturge–Weber Syndrome	Adrenal Adrenal functional adenoma/carcinoma Adrenal hyperplasia Congenital adrenal hyperplasia	Premature adrenarche
Acquired CNS lesion Tumors (Astrocytoma, optic glioma, craniopharyngioma, ependymoma) Post insults (Perinatal, trauma, infection, trauma, radiotherapy, chemotherapy) Cerebral palsy	Gonadotropin-producing tumors CNS chorioepithelioma, dysgerminoma, teratoma Teratoma, choriocarcinoma, hepatoma	
Genetic	Primary hypothyroidism Exogenous hormonal exposure	
Due to withdrawal of choric sex hormone exposure	Exogenous hormonal exposure	

**Table 2:** The role of diet in early life and during childhood on timing of puberty.

Period	Dietary Source	Mechanism of Action	BMI Dependent and/or Independent Action	Effect on Puberty	References
Early-life nutrition	Breastfeeding	Overweight prevention through normal hormonal and microbiome balance and positive psychosocial influence	Both	Precocious puberty prevention	[102,107,120,130,131,132,133,140,141,142,143,162]
	Formula feeding	Overweight development and predisposition to childhood obesity through increased IGF-1 and consequent enhanced sex steroid production	BMI dependent	Increased risk for precocius puberty	[136,153,154,158,159]
	Soy-based formulas	Weak estrogenic effects of soy isoflavones	BMI independent	Uncertain increased risk for precocius puberty	[166,167,171,172,173]
	Complementary feeding	Overweight development in case of age-inappropriate feeding and high protein consumption	BMI dependent	Increased risk for precocius puberty	[174,175,176,178,179]
	Soy-based foods	Weak estrogenic effects of soy isoflavones	BMI independent	Uncertain increased risk for precocius puberty	[166,172,180,181,186,187]
Childhood nutrition	High-energy diet	Higher levels of leptin, IGF-1 activation, adrenal androgen overproduction, and increased conversion of androgens to estrogens	BMI dependent	Increased risk for precocius puberty	[192,199,204,205,206]
	Macronutrients				
	Protein intake	Adiposity rebound before pubertal onset, IGF-1 secretion	Both	Increased risk for precocius puberty	[2,192,209,210]
	Fat intake	Direct effect on steroidogenesis and mammary gland development, indirect effect through induction of low-grade hypothalamic inflammation	BMI independent	Increased risk for precocius puberty (PUFAs). Uncertain increased risk for precocius puberty (MUFAs)	[2,192,217,221]
	Carbohydrate intake	Rapid increase in insulin concentration in high-glycemic-index diets resulting in increased availability of sex hormones and IGF-1	BMI independent	Uncertain increased risk for precocius puberty	[221,224]
	Micronutrients				
		Further studies are needed to identify the possible mechanisms	BMI independent	Uncertain increased risk for precocius puberty	[2,192,217,226]
	Dietary Pattern				
	Mediterranean diet	Reduction in circulating levels of estrogen, follicle-stimulating hormone, and luteinizing hormone. Increased excretion of estrogen. Stimulation of hepatic synthesis of SHBG, which reduces the biological availability of sex hormones	BMI independent	Precocious puberty prevention	[236]
	Vegetarian diet	Lower leptin levels	BMI dependent	Uncertain increased risk for later puberty	[238,239,240,242]
	New dietary patterns	A combination of the above mechanisms of high energy, fat, glycemic, and protein intake associated with unbalanced micronutrient supplies	Both	Increased risk for precocius puberty	[191,244,245]

## Data Availability

Not applicable.
